# Microbiota-Derived Metabolites Associated with Oats and Bran Attenuate Inflammation and Oxidative Stress via the Keap1-Nrf2 Pathway in Zebrafish

**DOI:** 10.3390/nu18020358

**Published:** 2026-01-22

**Authors:** Wen Duan, Tong Li, Yuyu Zhang, Baoguo Sun, Rui Hai Liu

**Affiliations:** 1 Key Laboratory of Geriatric Nutrition and Health, Beijing Technology and Business University, Ministry of Education, Beijing 100048, China; 15754367187@163.com (W.D.); zhangyuyu@btbu.edu.cn (Y.Z.); 2 Key Laboratory of Flavor Science of China General Chamber of Commerce, Beijing Technology and Business University, Beijing 100048, China; 3 Department of Food Science, Cornell University, Ithaca, NY 14853, USA; tl24@cornell.edu

**Keywords:** ursodeoxycholic acid, oxidative stress, inflammation, zebrafish, Keap1-Nrf2 pathway

## Abstract

Background/Objectives: Oats and oat bran are rich in polyphenols and soluble fiber, which are metabolized by gut microbiota into bioactive compounds. Previous studies identified ursodeoxycholic acid (UDCA), 3-(3-hydroxyphenyl)propionic acid (3-HPP), and avenanthramide C (AVC) as key microbial metabolites with protective effects against colitis. Methods: This study aimed to elucidate their antioxidant and anti-inflammatory activities and underlying mechanisms using LPS-induced RAW 264.7 macrophages and AAPH-induced oxidative stress in zebrafish embryos. All three metabolites significantly reduced intracellular reactive oxygen species (ROS), nitric oxide (NO), malondialdehyde (MDA), and pro-inflammatory cytokines (IL-6, TNF-α). They also restored mitochondrial membrane potential and enhanced superoxide dismutase (SOD) activity. Results:In vivo, treatment improved zebrafish survival, normalized SOD activity to 76–89% of control levels, and decreased ROS and MDA by 2.4 to 3.8 fold, with UDCA showing the greatest efficacy. Molecular docking revealed strong binding affinities to Keap1, particularly UDCA, which interacted with residues Met577, Ala440, Val532, and Val486. qRT-PCR further demonstrated downregulation of Keap1 and upregulation of Nrf2 and SOD, indicating activation of the Keap1-Nrf2 pathway. Conclusions: Collectively, these findings show that oats and bran-derived microbial metabolites exert potent antioxidant and anti-inflammatory effects via modulation of the Keap1-Nrf2 axis. Among the metabolites, UDCA exhibited the strongest biological activity at equivalent concentrations. This study provides mechanistic insight into how microbiota-derived oat metabolites contribute to redox balance and immune regulation, supporting their potential as functional components in dietary strategies for managing oxidative stress-related inflammatory diseases.

## 1. Introduction

Dietary interventions targeting the gut microbiota-host axis have emerged as a promising strategy for alleviating metabolic inflammation [1]. Oats (*Avena sativa* L.), a traditional cereal rich in bioactive compounds such as β-glucan, polyphenols, and avenanthramides, have demonstrated considerable anti-inflammatory and antioxidant potential in numerous studies. Our preliminary research confirmed that dietary supplementation with oats and oat bran significantly ameliorated dextran sulfate sodium (DSS)-induced colitis in mice by modulating the gut microbiota and enhancing short-chain fatty acid production [2]. Non-targeted metabolomic analysis of intestinal contents from mouse fecal samples, specifically from oats and oat bran, revealed that ursodeoxycholic acid (UDCA), 3-(3-hydroxyphenyl)propionic acid (3-HPP), and avenanthramide C (AVC) may act as key bioactive mediators underlying the health benefits of oat and oat bran interventions.

Increasing evidence suggests that the health benefits of oats not only from their native compounds but also from their gut microbial metabolites [3,4]. UDCA, a secondary bile acid produced by microbial metabolism, possesses anti-inflammatory, hepatoprotective, and antioxidant activities, and has shown therapeutic promise in inflammatory bowel diseases [5]. Similarly, avenanthramides, signature polyphenols in oats, especially AVC-exhibit significant antioxidant, anti-inflammatory, and antiproliferative effects, with potential roles in cancer prevention and inflammation control [6]. Studies have demonstrated that 3-HPP, a colonic metabolite of ferulic acid, can simultaneously extend lifespan and improve the health of Caenorhabditis elegans by reducing oxidative damage and enhancing stress resistance [7]. However, the precise mechanisms through which these metabolites exert anti-inflammatory and antioxidant effects remain insufficiently understood.

While in vitro studies are effective for revealing preliminary cellular mechanisms, they cannot replicate the complex systemic interactions and integrated responses of a whole organism. To bridge this gap, the zebrafish (Danio rerio) has become an increasingly valuable model organism due to its genetic similarity to humans (approximately 70% genome homology), high fecundity, short experimental cycles, and low maintenance costs [8,9]. Zebrafish models are particularly effective in studying oxidative stress, immune responses, and inflammation. For instance, 2,2′-azobis(2-methylpropionitrile) (AAPH) is widely used to induce oxidative stress in zebrafish embryos, facilitating the screening of antioxidant and anti-inflammatory agents and elucidation of their mechanisms of action [10,11].

Notably, the Keap1-Nrf2 signaling pathway is a central regulator of cellular antioxidant defense. Upon activation, Nrf2 translocates to the nucleus and upregulates the expression of various antioxidant enzymes, thereby playing a key role in mitigating oxidative damage and inflammatory responses [12]. Wu et al. [13] demonstrated that forsythoside B and alyssonoside exert anti-inflammatory effects by disrupting Keap1-Nrf2 interactions, promoting Nrf2 nuclear translocation, and enhancing the expression of downstream targets such as HO-1 and NQO1. Molecular docking further revealed that these phenylethanoid glycosides (PhGs) bind to Keap1 via hydrogen bonding, confirming their action through the Keap1-Nrf2-HO-1 signaling axis. However, it remains unclear whether ursodeoxycholic acid (UDCA), 3-(3-hydroxyphenyl)propionic acid (3-HPP), and avenanthramide C (AVC) exert their antioxidant and anti-inflammatory effects via this pathway. In particular, the molecular mechanisms underlying their potential interactions with the Keap1 protein warrant further investigation.

Therefore, this study aimed to investigate the anti-inflammatory and antioxidant properties of three oat-derived microbiota metabolites UDCA, 3-HPP, and AVC using both LPS-induced RAW 264.7 macrophages and an AAPH-induced zebrafish oxidative stress model. Through the integration of molecular docking, RT-qPCR, and oxidative stress biomarker analyses, we systematically evaluated their effects on inflammatory mediators, reactive oxygen species (ROS) scavenging, and antioxidant enzyme activity, with particular emphasis on the Keap1-Nrf2 signaling pathway as a potential mechanistic target. These findings offer new insights into the molecular mechanisms by which oat gut microbiota metabolites mitigate inflammation and oxidative stress, and support their potential use in the development of targeted dietary strategies for managing inflammatory conditions.

## 2. Materials and Methods

### 2.1. Materials and Reagents

Ursodeoxycholic acid was purchased from Beijing Solarbio Science & Technology Co., Ltd. (Beijing, China). Avenanthramide C was obtained from Sichuan Vicik Biotechnology Co., Ltd. (Chengdu, China) and 3-(3-Hydroxyphenyl)propanoic acid, along with E3 medium (5 mM NaCl, 0.17 mM KCl, 0.33 mM CaCl_2_, 0.33 mM MgSO_4_, and 0.1 μg/mL methylene blue) were obtained from Shanghai Macklin Biochemical Technology Co., Ltd. (Shanghai, China). Lipopolysaccharide (LPS) and tricaine (MS-222) were purchased from Sigma-Aldrich (St. Louis, MO, USA). The MitoTracker assay kit, nitric oxide (NO) assay kit, malondialdehyde (MDA) assay kit, and superoxide dismutase (SOD) assay kit were all obtained from Beyotime Biotechnology (Shanghai, China). Ripacell lysis solution was purchased from Beijing Solepol Technology Co. (Beijing, China). Dulbecco’s Modified Eagle Medium (DMEM), penicillin-streptomycin solution and fetal bovine serum (FBS) were obtained from Gibco (Thermo Fisher Scientific, Inc., Waltham, MA, USA). The JC-1 assay kit was purchased from Beyotime Biotechnology (Shanghai, China). Interleukin-6 (IL-6) and tumor necrosis factor-alpha (TNF-α) were acquired from Shanghai Enzyme-linked Biotechnology Co., Ltd. (Shanghai, China). The Evo M-MLV Reverse Transcription Kit was purchased from Accurate Biotechnology (Hunan) Co., Ltd. (Changsha, China).

### 2.2. Cell Culture and Viability

The RAW 264.7 macrophage cells were obtained from the American Type Culture Collection and cultured in high-glucose DMEM supplemented with 10% FBS and 1% penicillin-streptomycin solution. Cells were maintained at 37 °C in a humidified atmosphere containing 5% CO_2_ and subcultured regularly to sustain exponential growth. For viability analysis, cells were seeded into 96-well plates at a density of 1 × 10^4^ cells per well in 100 μL of medium and incubated for 24 h to allow adherence. Following this, cells were treated with varying concentrations of the test compounds for an additional 24 h. Cell viability was assessed using the MTT assay, and results were expressed as a percentage of the untreated control group.

### 2.3. NO Assay

RAW 264.7 cells in the logarithmic growth phase were harvested by centrifugation at 750× *g* for 5 min. The cell pellet was resuspended in complete culture medium (DMEM containing 10% FBS and 1% antibiotic/antimycotic) and adjusted to a density of 1 × 10^5^ cells per well, then seeded into plates and cultured until confluence. Cells were pretreated for 1 h with different concentrations of the test compounds: 250 µmol/L UDCA, 500 µmol/L 3-HPP, and 500 µmol/L AVC. Subsequently, lipopolysaccharide (LPS, 1 μg/mL) was added to induce inflammation, while the blank control group received an equal volume of culture medium.

After 24 h, 50 μL of culture supernatant was transferred to a 1.5 mL microcentrifuge tube and mixed with 50 μL of Griess reagent. The mixture was vortexed and incubated for 10 min at room temperature, protected from light. The absorbance was then measured at 540 nm using a microplate reader. NO production was quantified using a standard curve prepared with sodium nitrite [14].

### 2.4. ROS Assay

RAW 264.7 cells in the logarithmic growth phase were trypsinized, resuspended in culture medium at a density of 5 × 10^5^ cells/mL, and seeded into 96-well plates. After cell attachment, the medium was removed, and cells were pretreated with varying concentrations of UDCA, 3-HPP, or AVC for 6 h. Lipopolysaccharide (LPS, 1 μg/mL) was then added to induce inflammation, followed by a 24 h incubation.

After LPS stimulation, cells were incubated with 25 µmol/L DCFH-DA, a fluorescent probe for ROS detection, in the dark for 30 min. Excess probe was removed by washing three times with PBS. Intracellular ROS levels were visualized and imaged using an inverted fluorescence microscope [14].

### 2.5. Mitochondrial Membrane Potential Assay

The mitochondrial membrane potential was assessed using the JC-1 staining method, following the protocol described by Arranz et al. [15], with slight modifications. RAW 264.7 cells were seeded into 6-well plates at a density of 4 × 10^5^ cells/well in 2 mL of culture medium and incubated for 24 h to allow adherence.

Cells were then pretreated with UDCA, 3-HPP, or AVC for 6 h, followed by stimulation with 1 μg/mL lipopolysaccharide (LPS) for an additional 24 h. After treatment, the medium was aspirated, and 1 mL of JC-1 staining working solution was added to each well. Plates were gently mixed and incubated in the dark for 20 min. The staining solution was then removed, and cells were washed three times with JC-1 buffer. Finally, 2 mL of fresh culture medium was added to each well, and fluorescence images were captured using a fluorescence microscope.

### 2.6. Determination of Inflammatory Factors, SOD, and MDA Levels

Sample processing was conducted as described in Section 2.4. After treatment, culture supernatants were collected and centrifuged at 300× *g* for 10 min at 4 °C to remove cellular debris. The collected supernatant was used to measure IL-6 and TNF-α concentrations, following the manufacturer’s instructions.

For enzymatic activities analysis, cells were treated as described in Section 2.5, and lysates were subsequently prepared. Malondialdehyde (MDA) levels and superoxide dismutase (SOD) activity were determined using specific colorimetric kits (Beyotime Biotechnology, Shanghai, China) following the manufacturer’s instructions. The MDA assay was based on the thiobarbituric acid (TBA) reaction, while the SOD assay utilized the WST-8 method [16].

### 2.7. Zebrafish Rearing, Toxicity Assessment, and Establishment of an Oxidative Stress Model

Zebrafish experimentation was approved by Beijing Technology and Business University (BTBU-2025182). Wild-type AB strain zebrafish were purchased from the Chinese Academy of Fishery Sciences. Rearing conditions followed the standardized protocol described by Novichkova et al. [17]. Male and female zebrafish were housed separately under a 14 h/10 h light/dark cycle at 28 ± 0.5 °C in a ventilated recirculating aquaculture system and fed daily. After one month of acclimatization, fish were used for breeding.

Mating was conducted at a female-to-male ratio of 2:1. Embryos produced via natural mating under light conditions were collected and incubated in E3 medium. Fertilized embryos with normal morphology were selected under a stereomicroscope (Thermo Scientific, MA, USA) for subsequent experiments.

For toxicity evaluation, healthy embryos (*n* = 100–200) at 9 h post-fertilization (hpf) were randomly transferred into 24-well plates (15 embryos/well) containing 1 mL of E3 medium. Test compounds were administered at the following concentrations: UDCA (0, 5, 25, 50, 100, 250, and 500 µmol/L), AVC (50, 100, 200, 300, 400, and 500 µmol/L), and 3-HPP (0, 25, 50, 100, 150, 250, and 500 µmol/L). The sample size for this study was determined based on protocols established in the literature [18]. Each independent experiment used the same batch of fertilized embryos.

The medium was refreshed every 24 h. Embryo survival was monitored at 24, 48, 72, 96, and 120 hpf, and dead embryos were promptly removed. Each treatment group was performed in triplicate.

To induce oxidative stress, healthy embryos at 7–9 hpf were randomly distributed into 24-well plates (15 embryos/well) and treated with 25 mM AAPH until 24 hpf, as previously described by Kim et al. [18]. To minimize potential pain and distress, all live animal procedures were performed under MS-222 anesthesia. The zebrafish were closely monitored, and their swimming behavior was recorded. If any zebrafish exhibited severe physical deformities, tissue necrosis, or showed no response to gentle stimuli (such as light touch), rapid and humane euthanasia was carried out using an overdose of MS-222. Additionally, upon completion of the experiments, all healthy surviving larvae were returned to the aquaculture system.

### 2.8. Measurement of ROS in Zebrafish

Zebrafish embryos at 9 h post-fertilization (hpf) were randomly assigned to experimental groups. Fifteen embryos were placed in each well of a 24 well plate (*n* = 15/well) containing 0.5 mL of E3 medium. Treatment groups were pretreated for 1 h with 500 µmol/L ursodeoxycholic acid, 3-(3-hydroxyphenyl)propanoic acid, or avenanthramide C, while control groups received an equivalent volume of solvent.

Following pretreatment, all embryos were exposed to 25 mmol/L AAPH and incubated at 28.5 °C for 24 h to induce oxidative stress. Embryos were then transferred to fresh E3 medium and allowed to develop until 72 hpf. At that point, they were transferred to a black 96-well plate, stained with 25 μg/mL DCFH-DA in light-protected conditions, rinsed to remove excess dye, anesthetized with MS-222 (150 mg/L, pH 7.2), and immediately imaged using a fluorescence microscope.

### 2.9. Measurement of MDA and SOD Enzyme Activity in Zebrafish

Sample preparation was performed as described in Section 2.8. Zebrafish embryos were collected at 72 h post-fertilization (hpf), with 45 individuals per group. Embryos were rinsed with E3 medium and homogenized in pre-cooled phosphate-buffered saline (PBS) at a 1:10 (*w*/*v*) tissue-to-buffer ratio using a tissue disruptor. The homogenates were centrifuged at 12,000× *g* for 5 min at 4 °C, and the supernatants were collected for analysis.

Protein concentrations were determined using the bicinchoninic acid (BCA) assay. The methods for detecting SOD and MDA were consistent with those described in Section 2.6.

### 2.10. Molecular Docking Analysis

Molecular docking was performed using Kelch-like ECH-associated protein 1 (Keap1), a key regulator of the Nrf2 signaling pathway, as the receptor. The amino acid sequence of Keap1 was retrieved from the NCBI database, and the three-dimensional (3D) structures of UDCA, 3-HPP, and AVC were obtained from the PubChem database. Binding free energies were calculated to characterize the interactions between the ligands and the active site of Keap1. AutoDock Vina 1.2.0 was used to perform the docking simulations, following established protocols, including previously reported docking of TLB and XANA with the aryl hydrocarbon receptor (AhR). The best binding conformations were analyzed using Discovery Studio 2019 (DS 2019), and the ligand-protein complexes were visualized using PyMOL 1.7.2.1. Detailed procedures are described in Cai et al. [19].

### 2.11. Real-Time PCR

RT-qPCR was conducted to evaluate the effects of the three metabolites on inflammation-related gene expression in RAW 264.7 macrophages and antioxidant-related gene expression in zebrafish embryos. Total RNA was extracted using a commercial RNA extraction kit (Dongsheng, Guangzhou, China), and RNA purity was assessed with a NanoDrop 2000 spectrophotometer (Thermo Scientific, MA, USA). Complementary DNA (cDNA) was synthesized using a reverse transcription kit, following the manufacturer’s instructions.

For macrophage analysis, Glyceraldehyde-3-phosphate dehydrogenase (GAPDH) was used as the internal reference gene. The primer sequences were as follows: GAPDH (F: TGCGTGGCTTCCACACTTGCT, R: GAGGTCAATGAAGGGGTCGTT); TNF-α (F: CCTCTAGCCCACGTCGTAGC, R: AGCAATGACTCCAAAGTAGACC); IL-6 (F: GGCCTTCCCTACTTCACAAG, R: ATTTCCACGATTTCCCAGAG). For zebrafish, 45 embryos per group were collected for RNA extraction using trizol reagent. After reverse transcription, the expression levels of SOD, Nrf2, and Keap1 were quantified. The following primer sequences were used: GAPDH (F: GATACACGGAGCACCAGGTT, R: GCCATCAGGTACATACACGG); SOD (F: GTCGTCTGGCTTGTGGAGTG, R: TGTCAGCGGGCTAGTGCTT); Nrf2 (F: TTGTCTTTGGTGAACGGAGGT, R: CTCGGAGGAGATGGAAGGAAG); Keap1 (F: CCAACGGCATAGAGGTAGTTAT, R: CCTGTATGTGGTAGGAGGGTT). Relative gene expression was calculated using the 2^−ΔΔCt^ method.

### 2.12. Statistical Analysis

All data were analyzed using SPSS software (version 19.0). One-way analysis of variance (ANOVA), followed by Tukey’s post hoc test, was used to assess differences among groups. The data were plotted using Origin software (version 2021). A *p*-value < 0.05 was considered statistically significant. No animals, experimental units, or data points were excluded from the analysis for any of the experimental groups. Experiments were conducted in triplicate, and descriptive statistics (mean ± SD) were calculated for each group. Error bars represent the 95% confidence intervals.

## 3. Results and Discussion

### 3.1. Cytotoxicity and Concentration Selection of Metabolites

**Figure 1:**
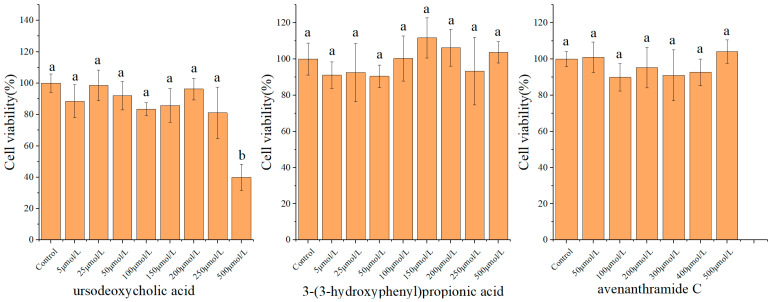
Effects of UDCA, 3-HPP, and AVC on the viability of RAW 264.7 cells. Different letters (a, b) indicate significant differences between groups (*p* < 0.05) as determined by one-way ANOVA.

The cytotoxic effects of UDCA, 3-HPP, or AVC on RAW 264.7 macrophages were assessed using the MTT assay. As shown in Figure 1, treatment with these metabolites at concentrations ranging from 5 to 250 μmol/L for 24 h resulted in cell viability exceeding 90%, indicating no significant cytotoxicity within this range. In contrast, exposure to 500 μmol/L of UDCA led to a marked decrease in cell viability (39.97 ± 8.30%), demonstrating substantial cytotoxicity at this higher concentration. Based on these results, the maximum non-cytotoxic concentrations-250 μmol/L for UDCA and 500 μmol/L for both 3-HPP and AVC were selected for subsequent experiments to ensure that any observed biological effects were not due to compromised cell viability. Moreover, this concentration was selected based on in vitro studies of natural products or gut microbial metabolites, aiming to address issues such as their low cellular uptake efficiency [20,21,22].

### 3.2. Effects of UDCA, 3-HPP, and AVC on ROS Levels and Mitochondrial Membrane Potential in RAW 264.7 Cells

**Figure 2:**
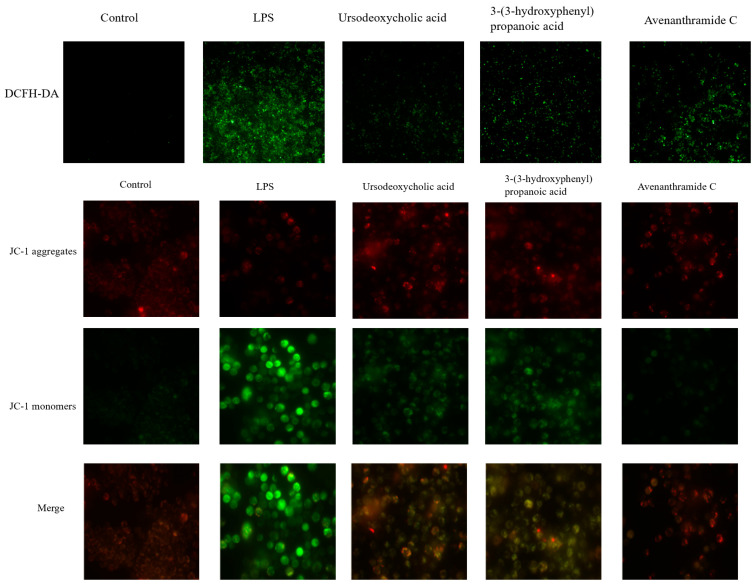
Effects of UDCA, 3-HPP, and AVC on ROS and mitochondrial membrane potentials. Representative fluorescent images of the ROS and representative fluorescent images of JC-1 staining showing the mitochondrial membrane potential.

Mitochondrial dysfunction and the resulting overproduction of reactive oxygen species (ROS) are key contributors to inflammatory activation [23]. As shown in Figure 2, lipopolysaccharide (LPS) stimulation induced significant oxidative stress in RAW 264.7 macrophages, as evidenced by increased green (DCF) and red (MitoTracker) fluorescence, indicating elevated intracellular and mitochondrial ROS levels. These observations are consistent with established models of inflammation-induced oxidative damage [24].

Treatment with UDCA, 3-HPP, and AVC significantly reduced ROS accumulation, with UDCA demonstrating the most pronounced antioxidant effect. This suggests that these metabolites can effectively counteract LPS-induced oxidative stress.

Further analysis using JC-1 staining revealed that LPS also caused a collapse in mitochondrial membrane potential (MMP), indicated by a fluorescence shift from red (JC-1 aggregates) to green (monomers). This loss of MMP, a hallmark of mitochondrial dysfunction and oxidative damage, plays a critical role in propagating inflammatory responses [17,25]. Notably, all three metabolites effectively restored MMP, indicating their capacity to preserve mitochondrial integrity under inflammatory conditions.

Collectively, these results suggest that the protective effects of these oat-derived metabolites, particularly UDCA are mediated through mitigation of ROS overproduction and stabilization of mitochondrial function. The superior efficacy of UDCA may be linked to its unique bile acid structure, which could facilitate direct interactions with mitochondrial membranes or redox-sensitive signaling pathways. These results indicate their capacity to preserve mitochondrial integrity under inflammatory conditions.

### 3.3. Effects of UDCA, 3-HPP, and AVC on NO, TNF-α, and IL-6 Production in RAW 264.7 Cells

**Figure 3:**
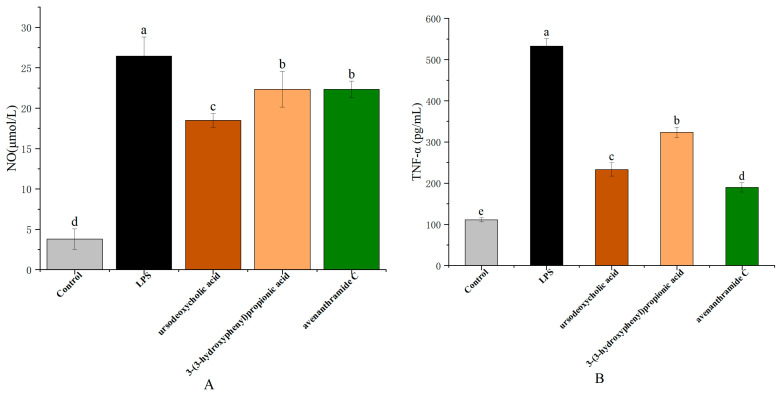

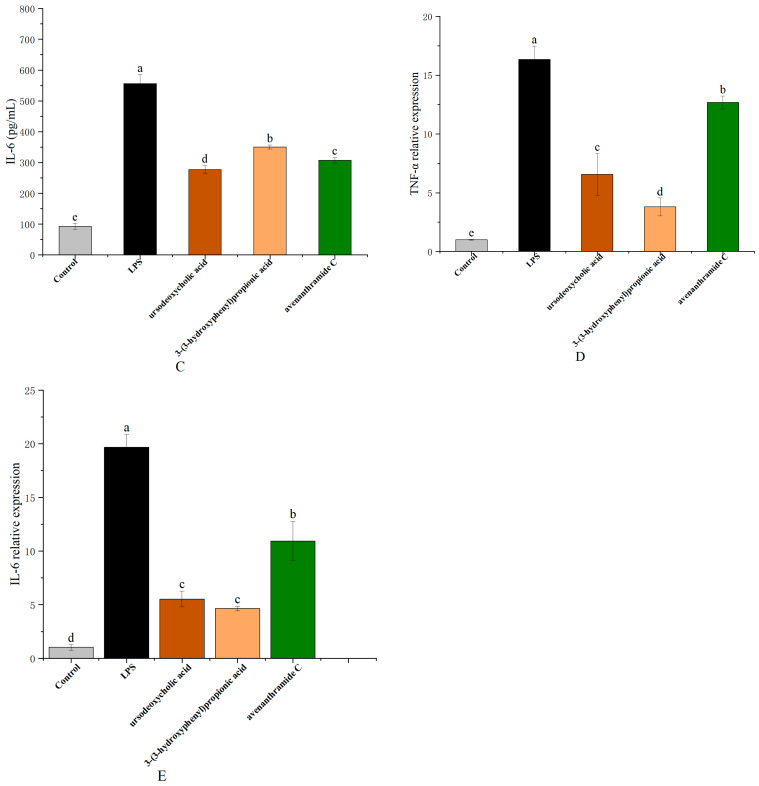
Effects of UDCA, 3-HPP, and AVC on LPS-induced production of (**A**) NO, inflammatory factors TNF-α (**B**), and IL-6 (**C**), and on the gene expression of TNF-α (**D**) and IL-6 (**E**) in RAW264.7 macrophages. Different letters (a, b, c, d and e) indicate significant differences between groups (*p* < 0.05) as determined by one-way ANOVA.

NO production in response to lipopolysaccharide (LPS) stimulation serves as a key marker of macrophage-mediated inflammation. As a critical signaling molecule, NO contributes to pathogen clearance and regulation of immune responses, thereby maintaining inflammatory homeostasis [26]. As shown in Figure 3, NO levels significantly increased from 3.80 ± 0.16 µmol/L in the control group to 26.45 ± 2.37 µmol/L following LPS treatment. Treatment with UDCA (250 µmol/L), 3-HPP (500 µmol/L), and AVC (500 µmol/L) significantly suppressed NO production to 18.48 ± 0.87 µmol/L, 22.35 ± 2.22 µmol/L, and 22.33 ± 1.00 µmol/L, respectively, with UDCA demonstrating the most pronounced inhibitory effect.

In addition to NO, pro-inflammatory cytokines such as tumor necrosis factor-alpha (TNF-α) and interleukin-6 (IL-6) are key mediators in the macrophage response to pathogenic stimuli. Polyphenols and related metabolites have been shown to modulate immune signaling pathways, affecting cytokine production and macrophage polarization. In line with these findings, treatment with UDCA, 3-HPP, and AVC significantly attenuated both the secretion and gene expression of TNF-α and IL-6. As illustrated in Figure 3, LPS exposure resulted in a 4.80-fold increase in TNF-α and a 5.98-fold increase in IL-6 levels compared to the control. The metabolites reduced TNF-α levels to 2.29-fold (UDCA), 1.65-fold (3-HPP), and 2.81-fold (AVC), and IL-6 levels to 2.01-fold (UDCA), 1.59-fold (3-HPP), and 1.81-fold (AVC), respectively.

These effects were further supported at the transcriptional level by RT-qPCR analysis, which showed that all three metabolites significantly downregulated LPS-induced expression of TNF-α and IL-6 mRNA (Figure 3). These results collectively indicate that the metabolites exert anti-inflammatory effects by inhibiting both the production and gene expression of key pro-inflammatory mediators [27].

Notably, the consistent efficacy of these gut microbiota-derived metabolites highlights the important role of dietary oat components in modulating immune responses. These findings provide mechanistic evidence supporting the anti-inflammatory potential of oat consumption and suggest that such metabolites may serve as promising dietary-derived therapeutic agents for inflammation-related conditions.

### 3.4. Effects of UDCA, 3-HPP, and AVC on Antioxidant Enzyme Activity and Lipid Peroxidation in RAW 264.7 Cells

The intracellular antioxidant defense system plays a vital role in maintaining redox homeostasis during inflammation [28]. To determine whether the anti-inflammatory effects of the tested metabolites were linked to enhanced antioxidant capacity, we evaluated the activity of superoxide dismutase (SOD), a key antioxidant enzyme, and the levels of malondialdehyde (MDA), a biomarker of lipid peroxidation and oxidative damage.

**Figure 4:**
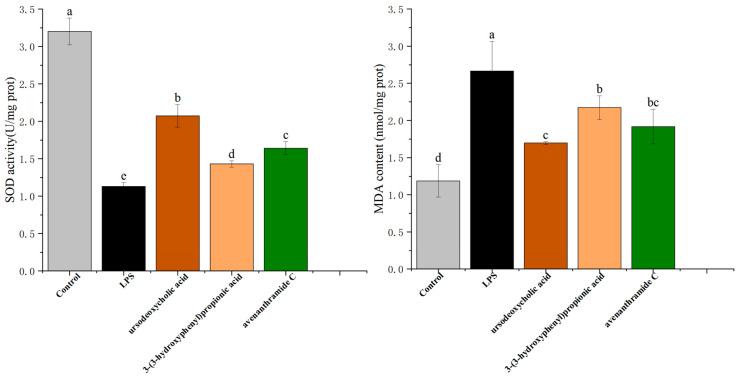
Effects of different concentrations of UDCA, 3-HPP, and AVC on LPS-induced SOD and MDA enzyme activities in RAW264.7 macrophages. Different letters (a, b, c, d and e) indicate significant differences between groups (*p* < 0.05) as determined by one-way ANOVA.

As shown in Figure 4, LPS stimulation significantly suppressed SOD activity, reducing it from 3.20 ± 0.18 U/mg protein in the control group to 1.13 ± 0.05 U/mg protein, indicating a marked impairment of the antioxidant defense system. Concurrently, MDA levels rose substantially under LPS-induced oxidative stress, reaching 2.67 ± 0.40 nmol/mg protein compared to control levels.

Treatment with UDCA (250 µmol/L), 3-HPP (500 µmol/L), and AVC (500 µmol/L) significantly reversed these effects. All three metabolites restored SOD activity, with UDCA exhibiting the strongest effect (2.07 ± 0.15 U/mg protein). Similarly, MDA levels were significantly reduced following treatment, particularly with UDCA, which lowered MDA to 1.70 ± 0.02 nmol/mg protein.

These findings suggest that the metabolites not only directly scavenge reactive oxygen species (ROS) but also enhance endogenous antioxidant defenses by upregulating SOD activity and reducing lipid peroxidation. The observed improvements in antioxidant enzyme function are consistent with the previous experimental results on ROS suppression and mitochondrial membrane potential restoration, reinforcing the conclusion that UDCA, 3-HPP, and AVC alleviate inflammation through regulation of oxidative stress pathways.

### 3.5. Toxicity Assessment of UDCA, 3-HPP, and AVC in Zebrafish Embryos

**Figure 5:**
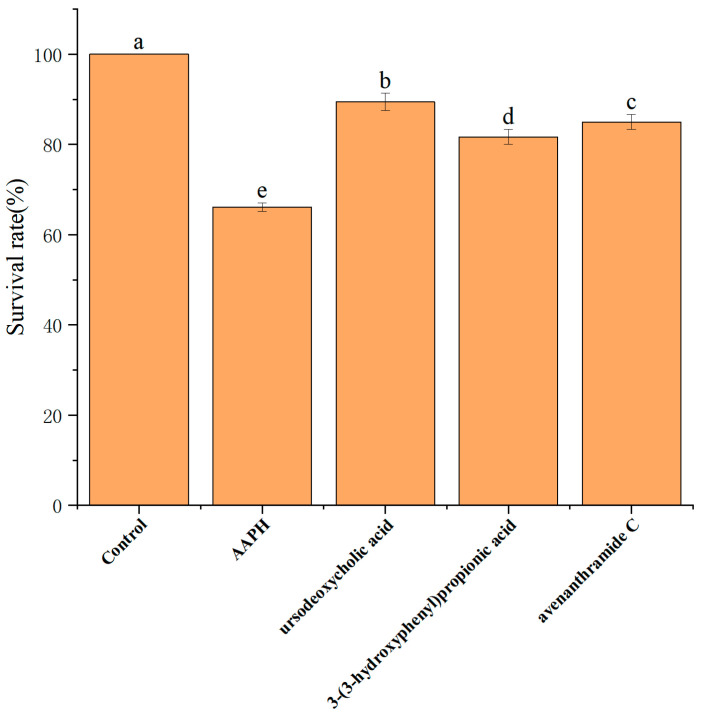
Survival of zebrafish embryos after co-treatment with 25 mM AAPH and three metabolites. Different lowercase letters (a, b, c, d and e) indicate significant differences between groups (*n* = 15 per group). Different letters (a, b, c) indicate significant differences between groups (*p* < 0.05) as determined by one-way ANOVA.

To investigate the in vivo safety and protective effects of UDCA, 3-HPP, and AVC, toxicity was assessed using a zebrafish embryo model. Under oxidative stress induced by 25 mM AAPH, embryo survival at 5 days post-fertilization (dpf) significantly declined to 65.0 ± 3.2% (Figure 5), indicating the severity of oxidative damage.

Administration of each metabolite at 500 µmol/L significantly improved survival rates, restoring embryonic viability to approximately 80–90% (Figure 5). Importantly, no signs of toxicity were observed at this concentration, confirming the compounds’ safety for in vivo use. Based on these findings, 500 µmol/L was selected as the working concentration for all three metabolites in subsequent zebrafish experiments.

### 3.6. Effects of UDCA, 3-HPP, and AVC on ROS Clearance and Antioxidant Activities in Zebrafish

Under immunocompromised conditions, excessive ROS can trigger lipid peroxidation, compromise membrane integrity, and impair receptor function. Oxidative stress disrupts the cellular redox balance, leading to structural damage and inflammation-associated pathologies [29]. To evaluate the antioxidant effects of UDCA, 3-HPP, and AVC in vivo, we assessed ROS levels using DCFH-DA staining and measured SOD activity and MDA content in zebrafish embryos.

**Figure 6:**
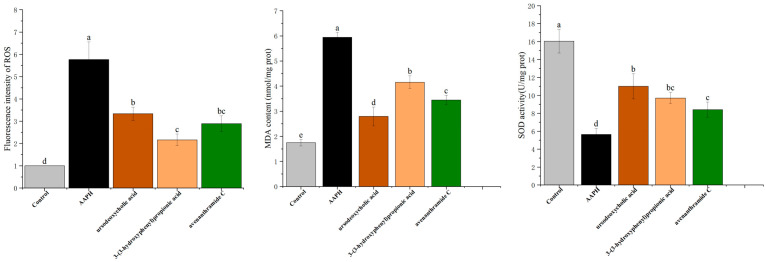
Effects of three metabolites on oxidative stress induced by 25 mM AAPH in zebrafish embryos. ROS levels (*n* = 15 per group), MDA enzyme activities and SOD enzyme activities (*n* = 45 per group). Different letters (a, b, c, d and e) indicate significant differences between groups (*p* < 0.05) as determined by one-way ANOVA.

As shown in Figure 6, AAPH-induced oxidative stress elevated ROS levels by 5.77 ± 0.42-fold compared to the control. Treatment with UDCA, 3-HPP, and AVC significantly reduced ROS accumulation by 3.61-, 2.41-, and 2.88-fold, respectively. Fluorescence imaging confirmed these reductions, illustrating the potent ROS-scavenging activity of all three compounds. These results are consistent with previous findings that phenolic compounds such as avenanthramide C can directly neutralize ROS [30].

ROS is known to activate nuclear factor kappa B (NF-κB), a central regulator of pro-inflammatory cytokine expression, thereby establishing a link between oxidative stress and inflammation. MDA, a byproduct of lipid peroxidation, serves as a reliable biomarker of oxidative damage [31]. As shown in Figure 6, exposure to AAPH resulted in a significant increase in MDA content compared to the control group. In contrast, administration of UDCA, 3-HPP, and AVC significantly attenuated MDA accumulation, although the extent of reduction varied among the treatments, with 3-HPP exhibiting the least pronounced effect in mitigating MDA levels.

These results align with clinical observations indicating that oat-derived phenolic compounds can enhance antioxidant enzyme activity and reduce lipid peroxidation markers such as MDA [6,32]. Overall, the three metabolites improved antioxidant defense by increasing SOD activity, reducing ROS and MDA levels, and mitigating oxidative damage in zebrafish, further supporting their protective roles in vivo.

### 3.7. Molecular Docking of UDCA, 3-HPP, and AVC with Keap1protein

To further explore the potential mechanism by which these microbiota-derived metabolites exert antioxidant effects, molecular docking simulations were conducted to assess their interaction with Kelch-like ECH-associated protein 1 (Keap1), a key regulator of the Keap1-Nrf2 pathway-recognized as a central modulator of cellular antioxidant defenses [33,34].

As presented in Table 1, all three metabolites exhibited favorable binding affinities to Keap1. UDCA displayed the strongest binding energy (−9.4 kcal·mol^−1^), followed by AVC (−8.7 kcal·mol^−1^) and 3-HPP (−6.2 kcal·mol^−1^). Binding energies below −7.0 kcal·mol^−1^ are indicative of high-affinity interactions, thus suggesting particularly strong interactions for UDCA and AVC.

**Figure 7:**
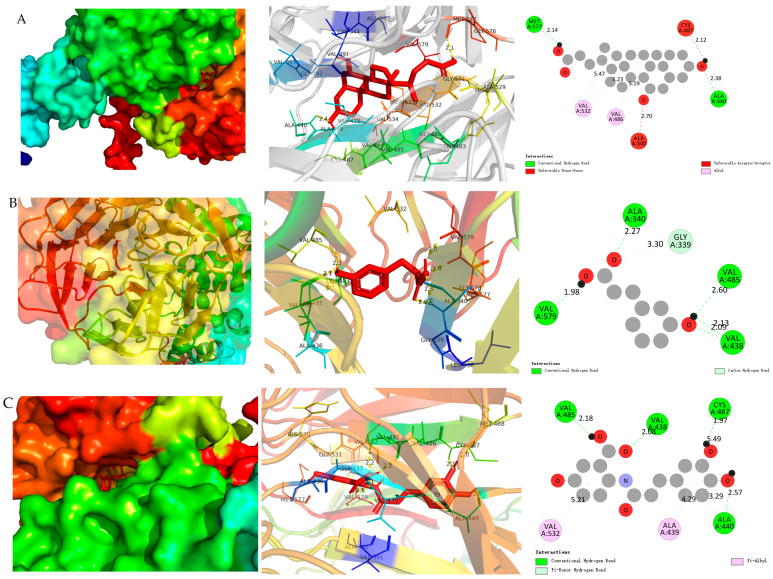
Molecular docking visualization of Keap1 protein by three metabolites. (**A**) ursodeoxycholic acid, (**B**) 3-(3-hydroxyphenyl)propionic acid and (**C**) avenanthramide C.

Detailed binding mode analysis revealed that UDCA forms hydrogen bonds with critical residues Met577 and Ala440, and engages in hydrophobic interactions with Val532 and Val486 (Figure 7). Similar interactions were observed for AVC and 3-HPP, albeit with lower binding strengths. These findings are consistent with previous reports showing that certain bile acids and phenolic compounds can activate the Keap1-Nrf2 signaling axis to protect against oxidative stress [35,36].

The superior binding affinity of UDCA aligns with its enhanced efficacy observed in both cellular and zebrafish models, supporting a structure–activity relationship in which Keap1 binding correlates with functional antioxidant outcomes. Taken together, these computational results, in conjunction with our in vitro and in vivo data, strongly suggest that activation of the Keap1-Nrf2 pathway constitutes a key mechanism through which oat-derived microbial metabolites alleviate oxidative stress and inflammation.

### 3.8. Effects of UDCA, 3-HPP, and AVC on Oxidative Stress-Related Genes Expression in Zebrafish Embryos

The Keap1-Nrf2 signaling pathway is a well-established regulator of cellular antioxidant defense and plays a critical role in protecting against oxidative stress-associated diseases [37]. To elucidate the molecular mechanisms underlying the antioxidant effects of the tested metabolites, we examined the expression of genes associated with this pathway in AAPH-induced zebrafish embryos using quantitative RT-PCR.

**Figure 8:**
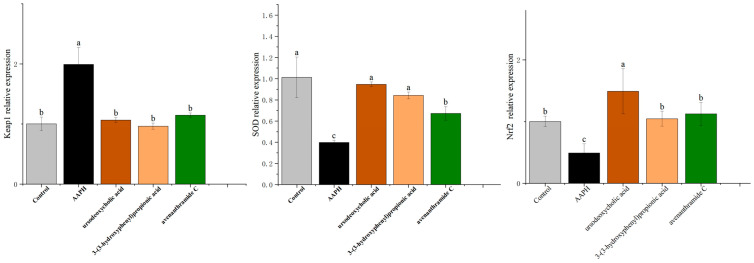
Effects of UDCA, 3-HPP, and AVC on SOD, Nrf2 and Keap1 gene levels in zebrafish embryos induced by 25 mM AAPH. (*n* = 45 per group). Different letters (a, b, c) indicate significant differences between groups (*p* < 0.05) as determined by one-way ANOVA.

As shown in Figure 8, AAPH exposure significantly downregulated the expression of Nrf2 and its downstream antioxidant target SOD, with transcript levels reduced by 2.04-fold and 2.54-fold, respectively, compared to the control group. This suppression reflects oxidative stress-induced disruption of the antioxidant signaling cascade.

Treatment with UDCA, 3-HPP, and AVC significantly restored the expression of both Nrf2 and SOD. UDCA exerted the strongest upregulatory effect on both genes, consistent with its superior antioxidant performance in previous assays [36]. In parallel, all three metabolites significantly suppressed Keap1 expression-by 1.87-fold (UDCA), 2.06-fold (3-HPP), and 1.73-fold (AVC)-thereby reducing repression of Nrf2 and facilitating its activation.

These gene expression patterns support coordinated modulation of the Keap1-Nrf2 pathway by the three metabolites. Our findings are consistent with prior studies demonstrating that polyphenolic compounds, including avenanthramides, activate Nrf2-mediated antioxidant defenses [30].

Together with the molecular docking and in vivo data, these results provide strong mechanistic evidence that microbiota-derived metabolites from oats alleviate oxidative stress by activating the Keap1-Nrf2 signaling axis. This supports the broader hypothesis that oat consumption contributes to systemic redox balance through microbial biotransformation into bioactive antioxidant compounds.

## 4. Conclusions

This study systematically evaluated the anti-inflammatory and antioxidant properties of three gut microbiota-derived metabolites UDCA, 3-HPP, and AVC whose production can be modulated by the gut microbiota in response to oats and bran consumption. The evaluation was conducted using LPS-stimulated RAW 264.7 macrophages and an AAPH-induced zebrafish oxidative stress model.

UDCA, 3-HPP, and AVC significantly suppressed the production of pro-inflammatory mediators, including NO, TNF-α, and IL-6, in LPS-activated macrophages. Additionally, they alleviated oxidative stress by reducing intracellular and mitochondrial ROS levels, restoring mitochondrial membrane potential, enhancing SOD activity, and decreasing MDA levels. In the zebrafish model, treatment with these metabolites improved survival under oxidative challenge and yielded consistent antioxidant benefits.

Molecular docking analysis revealed strong binding affinities between the metabolites and the Keap1 protein, suggesting activation of the Keap1-Nrf2 signaling pathway. This was further supported by gene expression data, which showed upregulation of Nrf2 and SOD, alongside downregulation of Keap1, indicating enhanced cellular antioxidant effects.

In summary, UDCA, 3-HPP, and AVC exert potent anti-inflammatory and antioxidant effects both in vitro and in vivo, primarily through modulation of the Keap1-Nrf2 axis. These findings highlight the potential of oat-derived microbial metabolites as promising dietary bioactives for the prevention and management of oxidative stress-related inflammatory diseases.

## 5. Future Perspectives

This study, employing both cell and zebrafish models, demonstrated that microbiota-derived metabolites from oats mitigate inflammation and oxidative stress through the Keap1-Nrf2 signaling pathway. However, given the significant differences in biological characteristics, physiological functions, and genetic backgrounds between humans and other species, these findings cannot be directly extrapolated to humans. In subsequent studies, we aim to further elucidate the dose–response relationships and mechanisms of action of these compounds by optimizing concentration gradients and including positive controls (e.g., dexamethasone). Additionally, we plan to employ an integrated approach, utilizing surface plasmon resonance (SPR), immunofluorescence, qPCR, and gene knockdown technologies to systematically validate this mechanism at the molecular, cellular, and functional levels. This will involve directly detecting compound-Keap1 interactions, observing Nrf2 nuclear translocation and downstream gene activation, and ultimately confirming the necessity of the pathway. Moreover, future research should focus on elucidating the interactions between specific oat components, such as β-glucan and polyphenols, and the gut microbiota. In particular, identifying the key microbial species and metabolic pathways responsible for the production of UDCA, 3-HPP, and AVC is essential. Such research will strengthen the role of oats as a preventive nutritional intervention against chronic inflammation.

## Figures and Tables

**Table 1:** Molecular docking of ursodeoxycholic acid, 3-(3-hydroxyphenyl)propionic acid and avenanthramide C with Keap1 protein.

Ligands and Receptors	Binding Energy	Amino Acid Binding Site (Distance)	
		Hydrogen Bonding	Hydrophobic Interaction
Ursodeoxycholic acid—Keap1	−9.4	Met 577 (2.14), Ala 440 (2.38)	Val 532 (5.47), Val 486 (5.19)
3-(3-Hydroxyphenyl)propionic acid—Keap1	−6.2	Ala 579 (1.98), Ala 340 (2.27), Val 485 (2.60), Val 438 (2.09), Gly 339 (3.30)	
Avenanthramide C—Keap1	−8.7	Val 485 (2.18), Val 438 (2.08), Cys 487 (1.97), Ala 440 (2.57)	Val 532 (5.21), Ala 439 (4.29)

## Data Availability

The original contributions presented in the study are included in the article. Further inquiries can be directed to the first author W.D. (15754367187@163.com).
